# Development and Implementation of a Tool to Assess Patient-Reported Outcome Measures (PROM) in Preoperative Setting

**DOI:** 10.17352/gjpm.000005

**Published:** 2017-11-16

**Authors:** Sunghye Kim, Pamela W Duncan, Leanne Groban, Hannah Segal, Rica Moonyeen Abbott, Jeff D Williamson

**Affiliations:** 1 Department of Internal Medicine, Wake Forest School of Medicine, Winston-Salem, NC, USA; 2 Sticht Center on Aging, Wake Forest School of Medicine, Winston Salem, NC, USA; 3 Department of Neurology, Wake Forest School of Medicine, Winston-Salem, NC, USA; 4 Department of Anesthesiology, Wake Forest School of Medicine, Winston-Salem, NC, USA; 5 Fisher Center for Hereditary Cancer and Clinical Genomics Research, Georgetown University, Washington, DC, USA

## Abstract

**Introduction:**

Traditional preoperative assessment tools use patients’ comorbidities to predict surgical outcomes, however, some functional, social and behavioral factors are known to predict surgical outcomes. Capturing functional, social and behavioral factors by incorporating patient reported measures (PROMs) into preoperative practice may be responsive to perioperative management and contribute to improved outcomes.

**Methods:**

We developed a preoperative PROM tool to identify functional, social, and behavioral factors. We describe the development and implementation of the tool as a health system quality initiative. We also report the results of the PROMs among preoperative surgical patients.

**Results:**

In our survey of 162 patients with mean age of 65, 53% were female, 29% were undergoing orthopedic surgery 12% were undergoing urologic surgery. 56% of the patients had at least one or more deficits in social or functional domain. The most common deficit was with ADLs with higher rate of deficit with advanced age.

**Conclusion:**

Implementation of a systematic assessment of functional and social determinants to improve processes of care in the preoperative setting is feasible. The majority of preoperative patients had at least one deficit and if identified preoperatively, appropriate interventions can be offered through well-designed intervention algorithms.

## Introduction

The US population is aging. While the individuals aged 65 and over made up only 11.2% of the US population in year 1980, it increased to 13% in year 2010 and it is expected to grow to 20.4% by year 2040 [1]. US healthcare systems observe the effect of this changing demographics of US population: patients who are ≥65 made up 38% of hospital discharges and 33% of all ambulatory surgeries in 2010 [2]. Advanced age is associated with increased postoperative complication, mortality, and functional status [3,4]. Traditional preoperative assessment tools use patients’ comorbidities [5,6], to predict surgical outcomes, however, some functional, social and behavioral factors are known to predict surgical outcomes [7–14], as well. Currently these factors are not usually collected in preoperative setting while some of the risk factors can be remediated preoperatively. Multiple survey questionnaires can be used to capture this information; however, a systematic approach might provide more effective and efficient way to obtain the information in preoperative setting. Patient Reported Outcome Measures (PROMs) are defined as any report of the status of a patient’s health condition that comes directly from the patient, without interpretation by a clinician or anyone else [15]. PROMs have been used to guide patient-centered care, clinical decision making, and health policy rulings, and are also an important tool for learning healthcare systems. PROMs improve communication between patient and providers as well as patient satisfaction [16,17] and they are well accepted by patients and clinicians [18]. PROMs may be used to improve perioperative care, by detecting preoperative deficits of patients as well as tracking postoperative trajectories. We describe the development of a preoperative PROM collection tool to identify the functional, social, and behavioral factors. We also report on the results of health system quality initiative of incorporating the PROMs tool into preoperative assessment clinic.

## Methods

Development of wake forest baptist health surgical pre-screen for optimal personalized care© (wfps), a tool for optimizing post surgery recovery

Based on literature review of the social and functional determinants and the utilization of PROMs, we identified 12 domains of social and functional factors and 1–4 questions for each domain that affect patients’ ability to self-management after discharge for optimal independence, health, and recovery (Table 1). With these domains, a health services researcher and physical therapist assembled and led an interdisciplinary team of clinicians (internist with geriatric experience, physical therapists, nurses, care coordinators, surgical service line leaders and health system leaders) to implement a PROM specifically developed to screen patients preoperatively. This PROMs tool, Surgical Pre-Screen for Optimal Personalized Care© was developed for use on an iPad- and linked to a web-based patient data collection platform (Tonic Health (tonicforhealth.com)).

The responses from the electronic assessment were available in real-time to generate a PDF report to the provider, care coordinators and patients. Reports included recommendations for postoperative care and discharge as well as alerts for significant risks that posed challenges for postoperative care management linked to algorithms (Table 2), for triggering needed services. We developed algorithms to generate recommendations for interventions as well as flag factors (e.g., challenges with medication management, i.e., skilled nursing facility) that could prolong recovery or contribute to poor outcomes. The algorithms identified impairments in a single domain (e.g. medication management) or they could also be combined throughout several of the domains of health to create a referral or identify community resources to meet the specific needs of the individual. For example the “Cognition,” “ADL “Social Support” and “Physical activity/Safe mobility’ deficits could accumulate or interact to create a recommendation for considering Skilled Nursing Facility or Assisted Living Placement for post-surgical recovery. The PROMs assessment tool was interviewer administered by a research assistant in the preoperative assessment clinic at Wake Forest Medical Center. The purpose of this implementation was to characterize the challenges that our patients may have, and to collaborate with care coordination to assess if PROMs implemented in our clinical setting would identify services preoperatively that the patient may need at hospital discharge to facilitate optimal recovery and independence, as well as to reduce readmissions and length of stay.

### Implementation of the tool in the preoperative assessment clinic

In the context of ongoing clinical care we used a convenience sample of 162 patients to administer the screening and assessments. Prospective participants were approached in the waiting room of the Preoperative Assessment Clinic, which serves >70 patients per day. We excluded those scheduled for emergency surgery. Once the primary visit and vital signs are completed by a clinic staff, a trained assistant interviewed the patient. The assistant interviewed all patients included in the study prior to their encounters with providers. Any deficiency with any of the questions in the short version would trigger the long version. The length of the interviews varied depending on the version: the short version took 3 minutes while long form took ten minutes on average. Following the assessment, the assistant filled out a “referrals recommended” page and informed the provider of any major concerns discovered during the interview. The assistant then met with a care coordinator to discuss the results from the questionnaire and the possible resources or referrals the patient may need. The coordinator would speak to the patient regarding possible referrals. A note was put into the patient’s electronic record of the visit if the coordinator cannot speak to the patient prior to the provider’s visit. The PROM interview did not delay the visit.

### Statistical analysis

Patients’ demographic variables, scheduled surgery, deficit in each domain were expressed as mean ±SD, or percentages as appropriate. Percentages of deficit by age category (≤50, 51–60, 61–70, 71–80, ≥81) were compared using Chi-Square tests. All statistical analyses were done with STATA/IC 14 (College Station, TX) and a 2-tailed test with p<0.05 was considered statistically significant.

## Results

Table 3 demonstrates characteristics and deficits of 162 enrolled patients. 67% of enrolled patients had at least one deficit. Among all the enrolled patients, the most common deficit was with ADL. Out of 91 patients who completed the long form that was triggered by any deficit in the short form, the most common deficit was fall risk.

**Figure 1:**
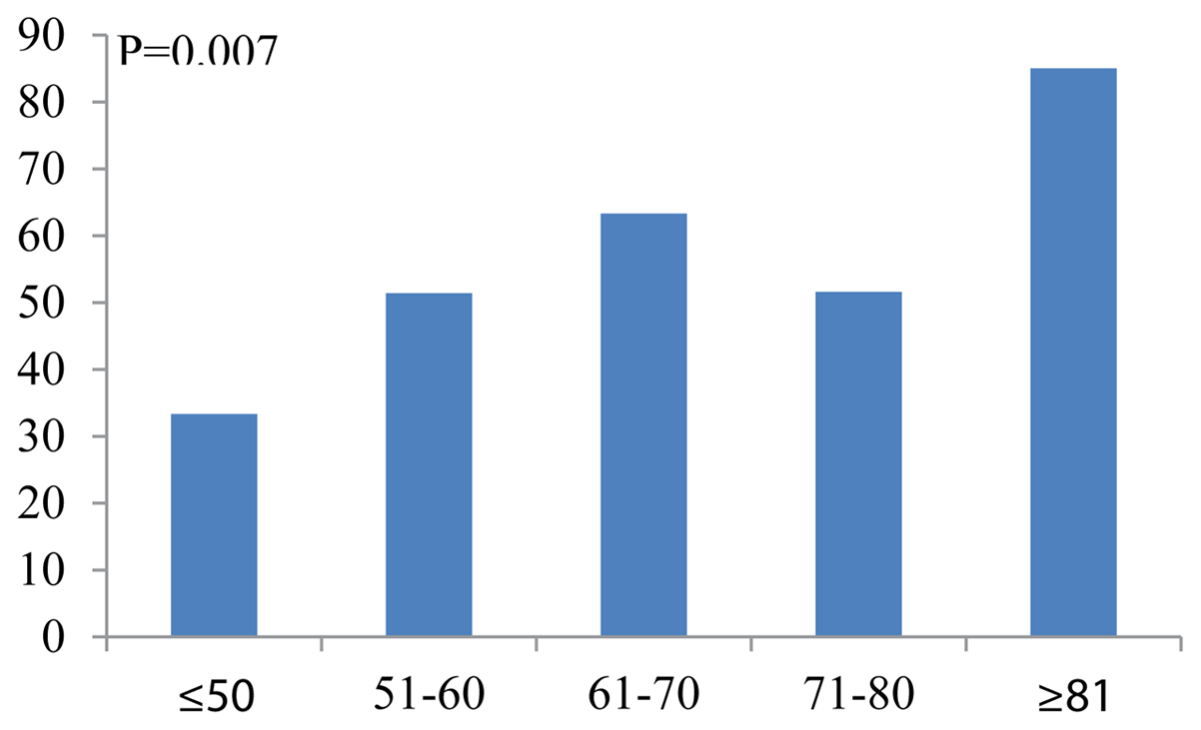
Proportion of patients with any deficits.

**Figure 2:**
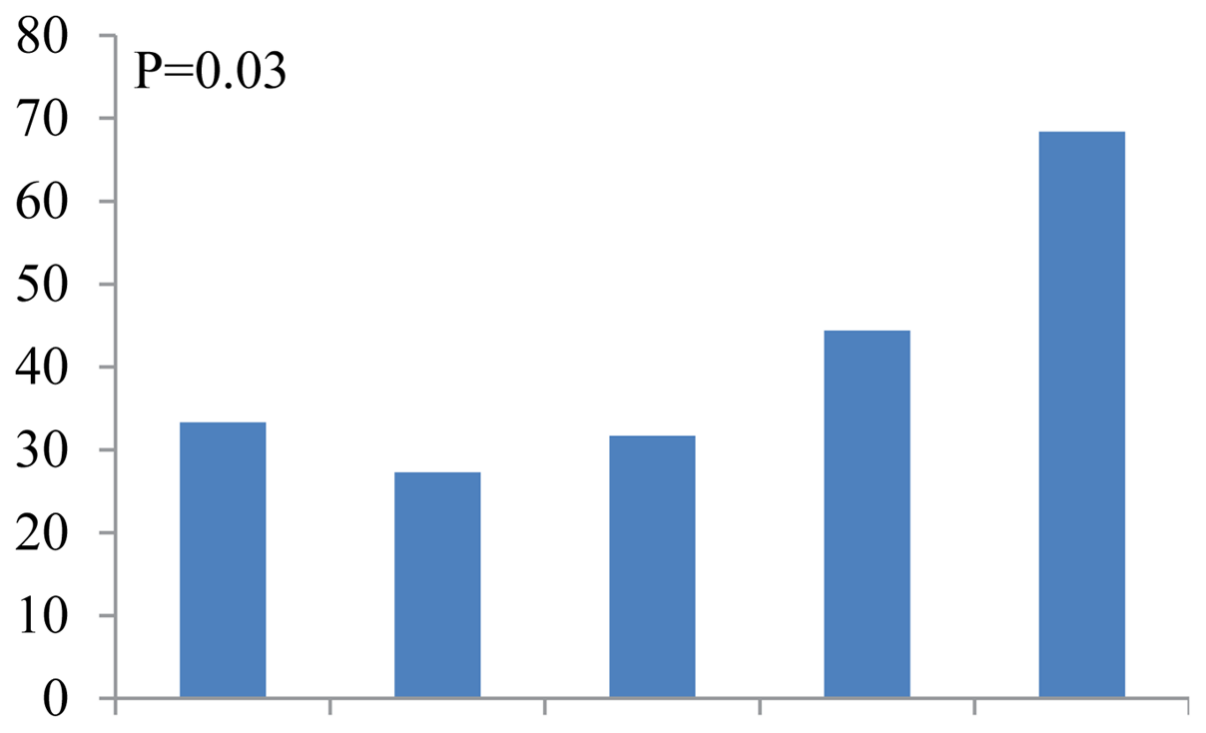
Proportion of patients with deficit in ADL.

Figures 1,2 demonstrates the proportion of patients with deficits with any deficits or deficit in ADL per age group. The proportion of patients with any deficit or deficit in ADL deficit was higher with advanced age.

## Discussion

Patients can provide critical information that can affect the postoperative outcomes and also can be intervened prior to surgery. Based on reviews of the social and functional determinants of surgical outcomes, we developed the Surgical Pre-Screen for Optimal Personalized Care©. This PROM assessed the major factors that could influence postoperative recovery, health and independence. Integration of the PROM data and well-designed electronic algorithms were used to identify and facilitate perioperative and post-discharge care needs in real time. In this quality improvement initiative, we demonstrated that if identified preoperatively, appropriate interventions can be offered through intervention algorithms. We also demonstrated that the implementation of the tool is feasible in the clinical workflow of a preoperative clinic setting. Preoperative PROMs have been developed and tested in certain surgery population [19–21]. However, most of the studies conducted the study in patients who are undergoing specific surgeries and assessed PROM in specific areas that the surgery will have impact (e.g., joint symptoms for joint replacement surgery). Our study enrolled patients who were undergoing various surgeries, including orthopedic, urologic, general, cardiothoracic, ophthalmologic, neurosurgery, breast or endocrine and endoscopic surgeries. We assessed preoperative their functional, social and behavioral factors and identified areas that the patients had deficits in. We found that more than half of the patients that we obtained PROMs had deficits in one or more domains. We did not find any association between the planned surgeries and deficits. The limitations of our study include the relatively small size of the sample and generalizability of the findings. We also did not measure surgical outcomes with this quality improvement project. Given the pilot nature of the study, we are planning on subsequent study that explore the surgical outcomes and correlate the outcomes with preoperative PROMs. We did not provide the opportunity for the patients to give us the feedback on the tool. With further study, we will incorporate patients’ feedback on the tool to improve the user friendliness and effectiveness of the tool.

## Conclusion

Efficient and effective assessment of the social and behavioral determinants of health, comprehensive assessment of functional status, health literacy, patient’s perception of health, and preferences for self-management may improve the success of postoperative management of vulnerable patients undergoing surgery and the Institute of Medicine recommended social and behavioral factors be incorporated into electronic health records as a pathway to improving care quality and safety (EHRs) [22]. In this quality improvement initiative, we demonstrated that if identified preoperatively, appropriate interventions can be offered through well-designed intervention algorithms. The implementation of the tool is feasible in the clinical workflow of a preoperative clinic setting.

## Figures and Tables

**Table 1:** 12 domains of PROMs.

Health Literacy	Tell me why you’re taking two of your medicines (No= +1) Can you tell me why you are having surgery? (No= +1)	1–2: red flag
**Medication Management**	Do you take more than 10 medicines a day? (>10/ Does not know=+1) Do you take more than 10 medicines a day? (>10/ Does not know=+1) Tell me why you’re taking two of your medicines (No= +1) When you feel better, do you sometimes stop taking you medicine? (Yes=+1) How often do you miss a dose of your medications? (1–3 times/week=+1) In the last month, were you unable to buy your medicines because of not having enough money? (Yes=+1)	1: red flag
**Cognition**	Please continue this sequence: 1,A,2,B,3,C… (No=+1) Please recall the three words I asked you to remember. (No=+1)	2:red flag
**Social Support**	If you were unable to walk without assistance for 30 days after your surgery, is there someone to help you get into your house, move about, get to the toilet or take a bath? (No=+1) If you are unable to drive after you operation for some period of time, is there someone who could take you to the doctor or pharmacy? (No=+1) Do you have currently have Home Health Care Service? (Yes= −1)	1–2: red flag
**Transportation**	Who is driving you home after surgery? (No one=1)	
**Primary Care Provider**	Do you have one doctor that knows you and all your medical conditions? (No=1)	
**ADL**	Can you get up out of a chair without using your hands? (No=1) Can you bathe/take a shower and dress yourself without any assistance? (No=1)	
**Physical Activity and Safe Mobility**	Can you walk without feeling unsteady? (No= +1) Can you go up and down 10 steps without help? (No=+1) Can you walk for at least 15 minutes without getting short of breath or needing to stop and rest? (No=+1)	
**Nutrition**	Do you eat at least 2 meals per day? (No=+1)	
**Depression**	Over the past two weeks, how often have you been bothered by little interest or pleasure in doing things (More than half/Nearly every day= +1) Over the past two weeks, how often have you been bothered by feeling down, depressed, or hopeless? (More than half/Nearly every day= +1	
**Falls risk**	Have you fallen in the last 6 months? (Yes= +1) Did you get injured and need to go to the doctor or ED? (Yes= +1) Have you fallen more than once in the last 6 months? (Yes= +1) Can you walk without feeling unsteady?	
**Financials**	In the last month, were you unable to buy your medicine because of not having enough money? (Yes=1)	

**Table 2:** An example of Medication Management Algorithm Flag.

Questions	If/Then Logic	Recommendation
Do you take more than 10 medications a day? (>10 =+1) (4–10=+2) (I don’t know = +2)	If>10=+2 If 4–10=+1 If “I don’t know”=+2	Score 3–5: Medication Management Red Flag “Patient is on multiple medication and may have some cognitive deficits with recall and sequencing or health literacy” “ If patients h as someone to help manage medications, there is no red flag regardless of recorded deficits”
Please continue this sequence: 1,A,2,B,3,C…	No=+1	
Please recall the three words I asked you to remember	No=+1	
Tell me why you’re taking two of your medicines	No= +1	
Does anyone help you manage your medications?	No=+1 Yes=−4	

**Table 3:** Characteristics of enrolled patients.

Characteristics	Result
Age (years, SD)	65 (±12.8)
Female (%)	53
Surgery planned (%)	
Orthopedic	29
Urologic	12
General	10
Cardiothoracic	7
Ophthalmologic	7
Neurosurgery	6
Breast or endocrine	6
Endoscopic	6
Other	17
Any deficits (n, %)	91(56)
Short form, Deficits (N, (%))	
ADL	53 (33)
Medication management	36 (22)
Cognitive impairment	28 (17)
Social isolation	14 (9)
Health literacy	8 (5)
No PCP	3 (2)
Long form, Deficits (N, (%))	
Fall risk	58 (64)
Depression	37 (43)
Financial	14 (15)
Nutrition	12 (13)
